# How safe are the biologicals in treating asthma and rhinitis?

**DOI:** 10.1186/1710-1492-5-4

**Published:** 2009-10-22

**Authors:** Linda S Cox

**Affiliations:** 1 Department of Medicine, Nova Southeastern University Osteopathic College of Medicine, Fort Lauderdale, Florida, USA

## Abstract

A number of biological agents are available or being investigated for the treatment of asthma and rhinitis. The safety profiles of these biologic agents, which may modify allergic and immunological diseases, are still being elucidated. Subcutaneous allergen immunotherapy, the oldest biologic agent in current use, has the highest of frequency of the most serious and life-threatening reaction, anaphylaxis. It is also one of the only disease modifying interventions for allergic rhinitis and asthma. Efforts to seek safer and more effective allergen immunotherapy treatment have led to investigations of alternate routes of delivery and modified immunotherapy formulations. Sublingual immunotherapy appears to be associated with a lower, but not zero, risk of anaphylaxis. No fatalities have been reported to date with sublingual immunotherapy. Immunotherapy with modified formulations containing Th1 adjuvants, DNA sequences containing a CpG motif (CpG) and 3-deacylated monophospholipid A, appears to provide the benefits of subcutaneous immunotherapy with a single course of 4 to 6 preseasonal injections. There were no serious treatment-related adverse events or anaphylaxis in the clinical trials of these two immunotherapy adjuvants. Omalizumab, a monoclonal antibody against IgE, has been associated with a small risk of anaphylaxis, affecting 0.09% to 0.2% of patients. It may also be associated with a higher risk of geohelminth infection in patients at high risk for parasitic infections but it does not appear to affect the response to treatment or severity of the infection.

Clinical trials with other biologic agents that have targeted IL-4/IL-13, or IL-5, have not demonstrated any definite serious treatment-related adverse events. However, these clinical trials were generally done in small populations of asthma patients, which may be too small for uncommon side effects to be identified. There is conflicting information about the safety TNF-alpha blocking agents, which have been primarily used in the treatment of rheumatoid arthritis, with serious infections, cardiovascular disease and malignancies being the most frequent serious adverse events. An unfavorable risk-benefit profile led to early discontinuation of a TNF-blocking agent in a double-blind placebo controlled of severe asthmatics.

In summary, the risk of anaphylaxis and other treatment-related serious events with of all of the biological agents in this review were relatively small. However, most of the clinical trials were done in relatively small patient populations and were of relatively short duration. Long term studies in large patient populations may help clarify the risk-benefit profile of these biologic agents in the treatment of asthma.

## Introduction

A number of therapeutic agents are available to treat the symptoms and inflammation associated with allergic rhinitis and asthma. Despite the proven efficacy of these medications, there continues to be some patients whose asthma [1] or rhinitis [2] is not well controlled. In addition, medications currently available for allergic rhinitis and asthma treatment appear to be are only effective while taken and do not appear to provide a sustained benefit after discontinuation [3]. Limitations of current medications and a greater understanding of the pathogenesis of allergic disease, has lead to the development of number of a novel therapeutic approaches as well as renewed interest in an old therapeutic approach, specific allergen immunotherapy. Novel therapeutic approaches that have been used in the treatment of allergic rhinitis and asthma include: omalizumab, alternate immunotherapy routes such as sublingual, modified allergen immunotherapy vaccines, anti-interleukin 5 (mepolizumab), interleukin-4 variant (pitrakinra) and tumor necrosis factor (TNF-α) blocking agents. The intent of this paper is to review the safety of these novel therapeutic approaches (see table 1 for summary of biological agents reviewed). Although the focus of the review is safety, there will be some discussion of the efficacy of these biological agents.

**Table 1:** Summary of biological agents used in the treatment of asthma and allergic rhinitis

Biological agent	Disease studied	Target	Mechanisms	Safety	Efficacy
SCIT	AR & asthma	Specific aeroallergens or venom	Several immune changes including↑IL-10 & TGF-β. isotype switch to IgG	Surveys suggest; fatality rate of 1 in 2.5 million injections^34-36 ^& near fatal reaction rate of 5.4 per 1 million injections^37^	Appears to depend on dose
SLIT	AR & asthma	Specific aeroallergens	Probably similar to SCIT	Most common AEs oral-mucosal symptoms AE less common than SCIT but cases of anaphylaxis have been reported	A consistent relationship with dose and efficacy has not been established^39^
MPL	AR	TRL4	Shift toward Th1 response	SRs reported in 1.6% of the 1736 patients in postmarking surveillence survey^50^	Clinical efficacy seen in first treat season after 4 injection treatment course
CpG	AR	TRL9	Shift toward Th1 response	No serious treatment-related effects^46^	Clinical efficacy seen in 1^st ^& 2^nd ^treatment season after one 4 injection course^46^
Omalizumab	Asthma & AR	IgE	Prevents binding of IgE to mast cells and basophils, downregulatuion of IgE receptor on these cells	Anaphylaxis in 0.09 to 0.2% of patients^19,20^	Efficacy in medication reduction & exacerbation in asthma, clinical improvement in AR
Mepolizumab	Asthma	IL-5	Blocks binding of IL-5 to α receptor on eosinophils	One episode of hypotension after infusion in EE study^57^	No significant improvement in asthma^55^
Pintrakinra^58^	Asthma	IL-4Rα receptor	Competes with IL-4 and IL-13 for binding to the receptor	Non-neutralizing IgG anti- pintrakinra antibodies in ~30% of pts	Increased PD_20 in_methacholine challenge & asthma AE & beta-agonist use
Etanercept	Asthma	TNF-alpha	soluble TNF-alpha receptor	No significant treatment-related AE is asthma ^59 ^but increased risk of serious & opportunistic infection in rheumatologic disease	Increased markers of TNF-alpha activity & improved clinical outcomes in refractory asthma ^59^

SCIT = subcutaneous immunotherapu, SLIT = sublingual immunotherapy, AR = allergic rhinitis, MPL = -deacylated monophospholipid A, CpG = immunostimulatory oligonucleotide sequence of DNA containing a CpG motif, TNF-alpha = tumor necrosis factor alpha, AE = adverse event, SAE = serious adverse event, EE = eosinophilic esophagitis, PD_20 _= Provocative Dose, which produces a decrease in FEV1 by 20% from the initial value or baseline value

### Definition and incidence of anaphylaxis

One of the difficulties in evaluating the safety of biologics is that some of the suspected adverse events may be related to the disease itself. For example, an increased risk of malignancy has been reported with TNF-alpha blockers, which are used primarily in the treatment of rheumatoid arthritis (RA). A systematic review and meta-analysis of 9 clinical trials that included 3493 RA patients treated with the anti-TNF-alpha antibodies, inflxumab or adalimumab, found a dose-related increase in malignancies compared with the control groups with a pooled odds ratio of 3.3 (95% confidence interval [CI], 1.2-9.1) [4]. This increased rate of malignancy was not seen in another study that compared the incidence of malignancy and cardiovascular disease in two large RA observational databases: BIOBADSER, a registry for safety of biologics, which included 4459 RA patients on TNF-alpha blocking agents in 100 centers who were followed from 2001 to 2006 and EMECAR, an external RA cohort (n = 789) established to define the characteristics of the disease and to assess comorbidity [5]. In the EMECAR registry, during the period of 1999 to 2005, a TNF-alpha blocking agent was given to 1.4%, 10.6% and 16.8% of the patients in 2000, 2003 and 2005, respectively. A higher incidence of malignancies was found in the EMECAR database patients, which included a minority receiving TNF-alpha blocking agents, compared with the BIOBADSER registry, in which all received a TNF-alpha blocking agents there was a higher incidence of malignancy in EMECAR vs. BIOBADSER (Relative Risk (RR) 2.9) and a lower BIOBADASER by EMECAR cancer-related mortality ratio, 0.36 (0.10-1.30).

Likewise, atopy and asthma have been identified as risk factors for anaphylaxis, the most severe and potentially life-threatening reaction associated with the biologics discussed in this review [6]. The incidence of anaphylaxis appears to be greater in the asthmatic population and this may be dependent on the severity of asthma. A review of a large patient database in the United Kingdom was performed to determine the incidence of anaphylaxis in the asthmatic and general population [7]. Specific Read codes were used to identify potential cases of anaphylaxis. All patients (10-79 years), who had at least one year of enrollment with a general practitioner and one health contact in the previous year during the period of 1996-2005 were included. Two cohorts were identified: asthmatic patients (791,225 person-yrs of follow-up) and the general population (884,745 person-years of follow-up). Within the asthmatic cohort, a subset of severe asthmatics, were identified based on meeting any of the following 4 criteria: '

1. ≥ 1 asthma hospitalization

2. ≥ 12 canisters of inhaled beta--agonists a year

3. ≥ 3 prescriptions of oral corticosteroids a year,

4. ≥ 3 classes of asthma medication a year

There were a total of 224 cases of anaphylaxis: 170 in the asthma population and 54 in the general population. The anaphylaxis incidence rate (cases/100,000 person-years) was highest in the severe asthmatic group: 43.1 in severe asthma patients, 15.4 in non-severe asthma patients and 6.1 in the general population. Compared to the general population, the age-gender adjusted RR for anaphylaxis was greater in both the severe asthmatic patients (RR 7.2, 95% confidence interval (CI), 5.0--10.3) and non-severe asthmatic patients (RR 2.5, 95% CI, 1.8--3.5). The authors concluded that there may be an increased risk of anaphylaxis in asthmatic patients that may be dependent on the severity of asthma.

Another variable and difficulty in interpreting the safety of biologics in research and postmarketing surveillance is that there is no universal agreement on the definition of anaphylaxis or the criteria for its diagnosis. In an attempt to resolve this problem, the National Institute of Allergy and Infectious Disease and Food Allergy and Anaphylaxis Network convened a meeting, which included representatives from multiple organizations and 3 continents, with the intent of developing a universally accepted definition of anaphylaxis and establishing clinical criteria that would accurately identify cases of anaphylaxis [8]. The working group developed three sets of criteria for the diagnosis of anaphylaxis (table 2). These criteria are used in some of the studies reviewed in this paper. However, in many studies and reviews, particularly ones involving allergen immunotherapy, the criteria for diagnosing anaphylaxis is not clear. Recognizing that adverse reactions to biological agents may be distinctly different from side-effects from chemicals and drugs, a classification system for adverse reactions to these agents has been proposed (table 3) [9]. This classification distinguishes five different categories:

**Table 2:** Clinical criteria for diagnosing anaphylaxis^8^

**Anaphylaxis is highly likely when any one of the following 3 criteria are fulfilled:**	1. Acute onset of an illness (minutes to several hours) with involvement of the skin, mucosal tissue, or both (e.g., generalized hives, pruritus or flushing, swollen lips-tongue-uvula)
**AND AT LEAST ONE OF THE FOLLOWING**	a. Respiratory compromise (e.g., dyspnea, wheeze-bronchospasm, stridor, reduced PEF, hypoxemia) b. Reduced BP or associated symptoms of end-organ dysfunction (e.g., hypotonia [collapse], syncope, incontinence)
**2. Two or more of the following that occur rapidly after exposure to a likely allergen for that patient (minutes to several hours):**	a. Involvement of the skin-mucosal tissue (e.g., generalized hives, itch-flush, swollen lips-tongue-uvula) b. Respiratory compromise (e.g., dyspnea, wheeze-bronchospasm, stridor, reduced PEF, hypoxemia) c. Reduced BP or associated symptoms (e.g., hypotonia [collapse], syncope, incontinence) d. Persistent gastrointestinal symptoms (e.g., crampy abdominal pain, vomiting)
**3. Reduced BP after exposure to known allergen for that patient (minutes to several hours):**	a. Infants and children: low systolic BP (age specific) or greater than 30% decrease in systolic BP* b. Adults: systolic BP of less than 90 mm Hg or greater than 30% decrease from that person's baseline

PEF = Peak expiratory flow; BP = blood pressure.

*Low systolic blood pressure for children is defined as less than 70 mm Hg from 1 month to 1 year, less than (70 mm Hg 1 [2 times age]) from 1 to 10 years, and less than 90 mm Hg from 11 to 17 years.

Sampson HA, Munoz-Furlong A, Campbell RL, Adkinson NF, Jr., Bock SA, Branum A, et al. Second symposium on the definition and management of anaphylaxis: summary report--Second National Institute of Allergy and Infectious Disease/Food Allergy and Anaphylaxis Network symposium. J Allergy Clin Immunol, 2006:391-7.

**Table 3:** Proposed classification of adverse side effects of biological agents^9^

Classification	Mechanism (s)	Clinical features
Type α	High cytokine & cytokine release syndrome	Symptoms will depend on the cytokine or cytokine being targeted e.g., high levels of INF-α may cause 'flu-like symptoms and anti- CD3 (muromunab) may induce cytokine release syndrome, which may include the following symptoms: flushing, arthralgias, capillary leak syndrome with pulmonary edema, encephalopathy, and severe gastrointestinal symptoms
Type β	Hypersensitivity	Immediate (IgE)
		Delayed (IgG or T cell)
Type γ	Immune or cytokine imbalance syndrome	Autoimmunity
		Allergic/atopic disorders
		Impaired function (immunodeficiency)
Type δ	Cross-reactivity	Will depend on the function of the cross-reacting antigen; e.g., Acneiform eruptions are commonly seen with cetuximab, an anti- epidermal growth factor receptor (EGFR) mAb possibly due to cross-reactivity between skin ERFR.
Type ε	Non-immunologic side-effects	Varies with the function of the biological agent; Interferon-α frequently associated with neuropsychiatric adverse effects

Type α reactions due to high cytokine levels, type β reactions due to a hypersensitivity reaction against the biological agent, type γ reactions due to immune or cytokine imbalance syndromes, type δ reactions due to cross-reactivity between the biological agent's target and cells that express similar or identical antigens and type ε reactions, which do not directly affect the immune system.

### Omalizumab

One of the most studied biologics for the treatment of asthma and rhinitis is omalizumab. Omalizumab is a 95% humanized monoclonal antibody (mAb) that binds to the Fc portion of the circulating immunoglobulin E (IgE) molecule preventing it from attaching to the high affinity IgE receptor, FcεR1. Omalizumab produces significant and rapid reductions in free serum IgE (up to 99%) [10]. One study demonstrated a 96% reduction in mean serum IgE level three days after omalizumab administration [11]. With continued treatment there is subsequent downregulation of the FcεR1 expression on several cell types that occurs over the next 4 to 6 months [12,13]. Omalizumab has been shown to be effective in the treatment of allergic asthma and seasonal and perennial rhinitis [14-17]. It is currently approved by the United States (US) Food and Drug Administration (FDA) for the treatment of moderate-to-severe persistent allergic asthma in adults and children 12 years and older. Omalizumab is also suggested as a treatment consideration for allergic asthmatics who are not well controlled with the combination of a medium-dose inhaled corticosteroid and a long-acting beta-agonist in the recently published expert panel guidelines on diagnosis and management of asthma [18]. These guidelines note that omalizumab is the only adjuvant therapy to demonstrate added efficacy to the combined regimen of a high-dose inhaled corticosteroid and a long-acting beta agonist [19].

The most common adverse reaction from omalizumab is injection- site pain and bruising but the package insert contains warnings regarding malignancies, geohelmith infections and a "black box" warning about anaphylaxis.

A "black box warning is the highest level of 5 possible warning categories found in pharmaceutical package inserts [20]. It is recommended, in the informed consent process, that patients be specifically informed that a medication has a "black box" warning and the reason for the "black box" warning be provided [20]. The "black box" warning on anaphylaxis (type β effect) was recently added to Xolair^® ^(omalizumab) package insert at the direction of the FDA in response to the spontaneous postmarketing adverse event-reports. A review of spontaneous postmarketing adverse events submitted to the FDA and Genentech/Novartis suggested that at least 0.2% of patients who received Xolair^® ^(omalizumab) experienced anaphylaxis between June 2003 and December 2006 [21]. The review also noted that many of the cases were delayed in onset and characterized by a protracted progression. An Omalizumab Joint Task Force of the American Academy of Allergy, Asthma and Immunology and the American College of Allergy, Asthma and Immunology (OJTF) also reviewed the postmarketing surveillance data from the same time period [22]. Using the definition of anaphylaxis proposed at a 2005 multidisciplinary symposia [8], the OJTF concluded that 35 patients had 41 episodes of anaphylaxis associated with omalizumab administration. During this time period 39,510 patients received omalizumab, thus the anaphylaxis-reporting rate was 0.09% of patients. Of those 36 events, for which the time of reaction was known, 22 (61%) reactions occurred in the first 2 hours after one of the first 3 doses and within 30 minutes in 5 (14%) of the events that occurred after the fourth or later doses.

The OJTF report recommends an observation period of 2 hours for the first 3 injections and 30 minutes for subsequent injections because 75% of the anaphylactic reactions occurred within these time periods. The OJTF report also provided recommendations on patient education regarding anaphylaxis.

### Omalizumab and malignancies

In the initial Xolair^® ^(omalizumab) clinical trials, a higher incidence of malignancies was observed in the group that received Xolair^® ^(omalizumab): 20/4127 (0.5%) Xolair^® ^(omalizumab)-treated patients compared with 5/2236 (0.2%) patients in the control group [23]. The observed malignancies in the Xolair^® ^(omalizumab)--treated patients were heterogeneous in tumor type and organ. There were no new cases of lymphoproliferative disease and no cases were considered drug-related by a panel of blinded independent oncologists. The majority of cases (60%) were diagnosed within 6 months of treatment [24]. Overall, the clinical data did not suggest a causal relationship between Xolair^® ^(omalizumab) and malignancy. However, the Xolair^® ^(omalizumab) package insert does note that "the impact of longer exposure to Xolair or use in patients at higher risk for malignancy (e.g., elderly, current smokers) is not known" [24]. A multicenter, prospective, observational cohort study titled Evaluating the Clinical Effectiveness and Long-Term Safety in Patients with Moderate to Severe Asthma (EXCELS) that includes approximately 5000 Xolair^® ^(omalizumab) -treated and 2500 with moderate to severe asthma not receiving Xolair^® ^(omalizumab), designed to evaluate the long term safety of Xolair^® ^(omalizumab) is currently in progress [25].

### Cardiovascular and Cerebrovascular disease

On July 16, 2009, the FDA issued a statement indicating that review of the EXCELS study's interim safety data showed an excess of cardiovascular and cerebrovascular events in the patients on Xolair^® ^(omalizumab) compared with the asthma control group [26]. The interim data submitted by the manufacturer, Genentech, suggests a disproportionate increase in ischemic heart disease, arrhythmias, cardiomyopathy and cardiac failure, pulmonary hypertension, cerebrovascular disorders, and embolic, thrombotic and thrombophlebitic events in patients treated with on Xolair^® ^(omalizumab) compared with the group that did not receive this medication.

Being an observational study, many factors need to be evaluated before determining the relationship between Xolair^® ^(omalizumab) and these adverse events. Considering that Xolair^® ^(omalizumab) is indicated for moderate- to-severe asthma and the prescribing/administration process can involve a fair amount of physician and staff time, as well as, patient time and expense, it is likely there were considerable differences between the Xolair^® ^(omalizumab)-treated group and the asthma control group in terms of asthma severity and co-morbidities. These differences may include oral corticosteroid use, underlying cardiovascular disease or hypertension and may account for the differences in adverse events. The FDA did not recommend any changes to the prescribing information (i.e., package insert) on Xolair^® ^(omalizumab) but did recommend that patients and physicians be aware that the new information from the EXCELS study may suggest a risk of crdiovascular and cerebrovascular events.

### Omalizumab and geohelminth infection

Considering that IgE may have a protective role in the immunity against parasitic infections, there are concerns that anti-IgE antibodies might impair this protective effect and increase susceptibility to parasite infection (type γ effect). The Xolair^® ^(omalizumab) package insert recommends that patients at high risk for geohelminth infections be monitored for such infections while on treatment citing a 1-year study of patients at high-risk for geohelminth infections, in which the odds ratio for infection in the Xolair^® ^(omalizumab)-treated group was1.96 with a 95% CI (0.88-4.36) compared with the placebo control. Similar results were seen in a 1-year double-blind, placebo-controlled (DBPC) trial of 137 subjects (12-30 years) at high risk of geohelminth infection, who were randomized to receive 52 weeks of treatment with Xolair^® ^(omalizumab) or placebo [27]. After adjusting for baseline infection status, gender, age and study visit, there was a significant difference in the incidence of intestinal geohelminth infection between the 2 groups (adjusted OR 2.2 95% CI; 0.94-5.15, p = 0.035:) However, there was no increased morbidity in terms of laboratory or clinical adverse events in the Xolair^® ^(omalizumab)-treated patients compared with the placebo group. The response to anti-geohelminth treatment was no different from the placebo group in both studies. These studies suggest that individuals at high-risk for geohelminth infections may be a greater risk for infection during Xolair^® ^(omalizumab) treatment but the infection severity and response to treatment is similar to placebo-treated patients.

### Allergen immunotherapy

Although this form of therapy is nearly 100 years old, it remains one of the only disease-modifying treatments for allergic asthma and rhinitis [28-30]. Subcutaneous immunotherapy (SCIT) with unmodified allergen extracts is currently the most widely used modality worldwide. However, sublingual immunotherapy (SLIT) has been prescribed with increasing frequency in the past 25 years, particularly in certain parts of Europe. SCIT efficacy appears to be dose dependent and the immunological mechanisms responsible for the clinical efficacy of immunotherapy are still being elucidated. Immunological changes associated with SCIT include induction of T regulatory cells, increase in allergen-specific IgG4, increase in IL-10 and TGF-β production and down-regulation of the TH-2 response [31]. Allergen immunotherapy has, been shown to decrease the recruitment of mast cells, basophils and eosinophils in the skin, nose, eye and bronchial mucosa, following provocation or natural exposure to allergens blocking both the immediate and late-phase allergic response [32]. Adverse reactions from SCIT include common local injection site reactions to rare life-threatening anaphylaxis and death. The frequency of systemic reactions with SCIT appears to be related to the schedule and dose. In one review of 38 SCIT studies the systemic reaction (SR) rate with conventional build-up schedules (single dose increase per visit) ranged between 0.05% to 3.2% of injections and 0.8% to 46.7% of patients (mean,12.92%) [33]. In a 1-year prospective study of a multi-physician practice, there were 98 SRs in 96/4578 (2%) patients [34]. There was a significant difference in per injection SR rate between the maintenance (1 per 1831 visits) and build-up phase (1 per 1063 visits, p = 0.01).

Accelerated immunotherapy build-up schedules for inhalant allergens may be associated with greater risk, although this has not been consistently demonstrated with cluster schedules (2 to 3 injections per visit on non-consecutive days). A recent review of accelerated immunotherapy schedules found the SR rate for rush immunotherapy (multiple injections given over 1 to 3 days) ranged from 15% to 100% of patients without premedication and 14.7% to 38% of patients with premedication [35]. A similar range was seen with cluster schedules, although one study specifically designed to compare the safety of a cluster schedule with a conventional schedule found no significant difference in the SR rate between the groups and the clinical and objective parameters improved 6 weeks sooner in the cluster group [36].

Severe reactions from SCIT are relatively rare but fatal reactions (FR) have been reported at a rate of 1 in 2 to 2.5 million injections or an average of 3.4 deaths per year in surveys of AAAAI membership that span the time period from 1945 to 2001 [37-39]. Symptomatic or poorly controlled asthma has been identified as a risk factor for a FR from SCIT. The most recent survey also assessed the frequency of SCIT near-fatal reactions (NFR) defined as severe respiratory compromise and/or fall in blood pressure requiring emergency treatment with epinephrine [40]. The incidence of unconfirmed NFRs was 23 per year or 5.4 events per one million injections. Important contributing factors for NFR included administration during peak of the allergy season (46% of respondents) and dosing errors (25% of respondents) Fifty-eight percent of the NFR received injections from the maintenance concentrate.

### Sublingual immunotherapy

One of the purported advantages of SLIT over SCIT is greater safety. Like SCIT, the mechanisms of SLIT are still being elucidated but studies have suggested similar immunological changes with SLIT [41]. A number of studies have demonstrated the efficacy of SLIT in the treatment of allergic rhinitis and asthma, but the determinants of SLIT efficacy have not been clearly established. There did not appear to be a consistent relationship between dose and SLIT efficacy in a comprehensive review of 104 articles on SLIT [42]. In this review, there were 66 studies that provided some information on SLIT safety. In these 66 studies, there were approximately 1,181,000 doses given to 4378 patients. There were no fatalities or events described as anaphylaxis, although there were 14 probable serious adverse events (7 were asthma reactions). Oral-mucosal reactions, considered a SLIT local reaction, were relatively common, affecting up to 75% of patients, and seen most frequently in the build-up phase. In the studies that specified the type of reaction, 169 of 314,959 (0.056% of doses administered) were classified as SRs There were 244 moderate adverse events (AE) requiring dose adjustment or causing withdrawal from the study in 2939 patients treated for 4586 treatment years with 810,693 doses of SLIT (50 studies). The majority of these reactions were gastrointestinal symptoms, rhinoconjunctivitis, urticaria or some combination of these symptoms. There were no identified risk factors for SLIT adverse reactions in this review. There did not appear to be a consistent relationship between adverse reactions and SLIT dose or induction schedule and the majority of studies were done in rhinitis/rhinoconjunctivitis patient with or without mild-to-moderate asthma. There were no studies in high risk patients (e.g., moderate to severe asthmatics).

Subsequent to this review, there have been several case reports of SLIT-associated anaphylaxis:

• One occurred on the 3^rd ^day of build-up with a multi-allergen SLIT [43]

• One occurred on maintenance at the height of season [44]

• One occurred on the 4^th ^day of a latex rush protocol [45].

• One occurred after a 3 week gap in maintenance treatment after taking a dose 6 times higher than prescribed. This reaction resulted in loss of consciousness and admission to the intensive care unit [46].

Four cases of SRs with SLIT have been reported in individuals who had to discontinue SCIT due to a SR [47,48]. Two cases occurred after the first dose of a grass tablet [48]. The other two cases occurred with the maintenance dose of SLIT administered as drops shortly after completing an ultrarush protcocol [47].

Some of the factors in the above cases are risk factors that have been identified with SCIT: height of season, history of prior SRs, dose and schedule. Further studies are needed to identify and characterize SLIT risk factors.

### Modified allergen vaccines and adjuvants

Efforts to develop safer and more effective allergen immunotherapy vaccines have resulted in several modifications to the allergen extracts. Allergoids are modified allergen extracts that have been processed in a way that reduces the extract's allergenicity while preserving its immunogenicity. Adjuvants have also been used to enhance the effectiveness of allergen immunotherapy primarily by shifting the immune response toward Th1 production. The two adjuvants that have been most extensively studied in the treatment of allergic rhinitis, are an immunostimulatory oligonucleotide sequence of DNA containing a CpG motif (CpG) and 3-deacylated monophospholipid A (MPL), both of which target toll-like receptors (TLR). Toll-like receptors play a key role in activating antigen-presenting cells and when stimulated can influence the Th1/Th2 cytokine balance. The receptor for CpG DNA is TLR9, which is primarily expressed on plamacytoid dendritic cells [49]. Activation can lead production of IL-10, IgG isotype switching and other inhibition of other immune responses mediated by Th2 cells. TOLAMBA, a TLR9 agonist, is a CpG adjuvant that is covalently linked to the major ragweed allergen Amb a 1. A randomized DBPC, phase 2 trial of 25 adults with ragweed-induced allergic rhinitis randomized to receive 6 increasing doses of TOLAMBA (0.06, 0.3,1.2, 3.0, 6.0 and 12 mcg) or placebo before the ragweed season and followed through 2 ragweed seasons found no "pattern of vaccine-associated systemic reactions or clinically significant laboratory abnormalities" [50]. Although, there was no difference in the primary outcome, which was albumin level in nasal-lavage fluid after nasal allergen provocation, there was a significant reduction in total nasal symptom scores during the peak season in the TOLAMBA group compared with the placebo-treated patients in both the first and second ragweed season. A subsequent placebo-controlled trial of 738 subjects with ragweed-induced allergic rhinitis who were randomized to either a high dose regimen (TOLAMBA 3,9,30,30,30, 30 mcg), a low dose regimen (TOLAMBA 1,2,3,6,15,21, 30 mcg) or placebo reported that treatment "was well tolerated in all groups" and that there were no TOLAMBA-related serious adverse events [51]. On January 8, 2007 Dynavax Technologies Corporation announced that the analysis of interim one-year data from this two-year study (DARTT trial) indicated that there was "no measurable disease during the ragweed season in any of the study groups making it impossible to measure the therapeutic effect of TOLAMBA treatment and the study" [52]. Based on these results, Dynavax Technologies decided to discontinue development of TOLAMBA.

MPL, the other adjuvant used in allergen immunotherapy is derived from the lipopolysaccharide of *Salmonella Minnesota *R595. It contains lipid A, which is a TLR4 agonist, that has been has been shown to induce TH1 cytokines in human and animal studies. An allergen vaccine composed of a tyrosine-absorbed (delays absorption), glutaraldehyde-modified allergen (allergoid) containing the MPL adjuvant has been shown to provide significant improvement in clinical and immunological parameters in patients with seasonal rhinitis [53-55]. The dosing regimen was 4 doses (300,800, 2000, 2000 standardized units (SU) administered at 1-2 week intervals ending 2 to 4 weeks before the start of season. The highest dose and cumulative dose was equivalent to 24 mcg and 60 mcg of group 1 grass pollen allergen, respectively [53]. The treatment was well tolerated in the clinical trials with systemic adverse events occurring at a similar frequency in the active and placebo groups [53,56,57]. There were no serious or severe adverse events or anaphylactic reactions in these clinical trials. In a one-year postmarketing surveillance assessment of 1736 patients, who received a total of 8512 injections, systemic reactions were reported by1.6% of the patients [54]. Fourteen patients reported severe reactions but there were no instances of anaphylactic shock. One of the potential adverse effects of concern with Th1-inducing adjuvants is autoimmunity (type γ effect). In addition to the clinical immunotherapy trials, MPL has been used as an adjuvant in licensed vaccines for many years: Melacine^® ^(Corixa Inc./Schering-Plough, Canada) and the human papillomavirus, Ceravarix^® ^(Glaxo-Smith-Kline, UK). To date, there has been no evidence of increased incidence of autoimmune diseases in the populations that have been exposed to MPL

In the past four years, there have been several clinical trials designed to study the efficacy and safety of MPL in the treatment of grass-pollen or ragweed-induced allergic rhinitis (Grass MATAMPL, RagweedMATAMPL, Allergy Therapeutics Ltd.) conducted in the US and Canada. However, the FDA placed a clinical hold on all of MPL-related vaccine studies in the US after a case of transverse myelitis was reported in the grass-pollen study.

### Mepolizumab (anti-IL-5)

Mepolizumab is a high-affinity, humanized non-complement-fixing IgG_1_mAb that blocks binding of IL-5 to the α chain receptor complex on eosinophils. IL-5 plays key role in eosinophilic differentiation, maturation, migration, activation and survival and it is highly expressed in bronchial alveolar lavage fluid and biopsies of asthmatics. Single dose and multiple dose (6 monthly) intravenous and subcutaneous mepolizumab toxicity studies in monkeys found no target organ toxicity or immunotoxicity with doses up to 300 mg/kg [58]. In a DBPC study of 363 asthmatics, symptomatic on inhaled corticosteroids, randomized to 3 infusions of either placebo, 250 mg or 750 mg of mepolizumab, there were no significant differences between the 3 groups in any of the clinical outcomes [59]. However there was a trend toward reduced exacerbations in the 750 mg dose group (p = 0.065). Side effects were similar in the three groups and there were no serious adverse events attributed to the study medication. The withdrawal rate due to adverse reactions was highest in the placebo group: placebo (5%), 250 mg (3.3%) and 750 mg 0.9%). Mepolizumab has also been used is small open-label studies of patients with eosinophilic disorders. In a study of 4 patients with hypereosinophilic syndrome, who received 3 infusions of up to 750 mcg of mepolizumab, there was a reduction in peripheral eosinophils and clinical improvement in all 4 patients [60]. The only reported medication- related AEs were fatigue and headache after the infusions. In another open label study of 4 patients with eosinophilic esophagitis treated with the same protocol, there was one episode of symptomatic hypotension 30 minutes after the 3^rd ^infusion, which resolved with fluid replacement [61]. The investigators questioned whether this was related to the infusion. Other symptoms reported in this trial included nausea, fatigue, headache, non-specific chest pain and cough. All patients reported improved clinical outcomes.

Essentially, there was one possible serious-adverse event (hypotension) and no evidence of cytokine imbalance syndrome (type γ effect) in the relatively small populations of patients in these mepolizumab clinical trials.

### Interleukin-4 and Interleukin-13 Inhibition

Interleukin-4 (IL-4) and Interleukin-13 (IL-13) are cytokines that play a role in allergic inflammation by inducing Th2 responses. Interleukin-4Rα is the signaling component of the receptor complex for both IL-4 and IL-13. Pitrakinra is a recombinant IL-4 variant that competitively binds IL-4Rα receptor inhibiting the binding of both IL-4 and IL-13. Two DBPC 28 day trials of pitrakina administered subcutaneously (SQ) or via nebulizer demonstrated some clinical efficacy [62]. There was a significant difference in post allergen challenge FEV_1 _compared with the placebo group in the SQ pitrakinra study and fewer asthma-related adverse events and beta-agonist rescue use in the group that received inhaled pitrakinra. There were also no significant differences in safety outcomes between the pitrakinra and placebo groups except for an increased frequency of injection site reactions in those who received SQ pitrakinra. IgG antibodies against pitrakinra were seen 3/10 patients in the SQ study (titers of 1:40 and 1:80) and 3/15 patients in the inhalation study (titers were1:30, 1:60, and 1:480). None of IgG antibodies against pitrakinra were able to block the binding of the drug to interleukin 4Rα; i.e., they were non-neutralizing antibodies.

### Tumor Necrosis Factor Alpha Blocking Agents

TNF-alpha blocking agents have been used extensively in rheumatology primarily in the treatment of rheumatoid arthritis. One randomized DBPC, cross-over study investigated the effects of ten-weeks of treatment with etanercept, a soluble TNF-alpha receptor, administered twice weekly to 10 patients with refractory asthma [63]. Etanercept was associated with a significant increase in the methacholine provocation dose (P = 0.05), an improvement in the asthma-related quality of life scores (by 0.85 point; P = 0.02), and a 0.32-liter increase in post-bronchodilator FEV_1 _(p = 0.01). There were no treatment-related adverse events or withdrawals. The study also measured markers of TNF-alpha activity and found that patients with refractory asthma had increased expression of membrane-bound TNF-alpha, TNF-alpha receptor 1, and TNF-alpha-converting enzyme by peripheral-blood monocytes compared with the mild-to-moderate asthma and the control groups. This study suggests that patients with refractory asthma may have up-regulation of the TNF-alpha axis and may benefit from treatment with a TNF-alpha blocking agent.

In a double-blind placebo controlled trial 309 patients with severe, uncontrolled asthma randomized to one of 3 doses of the TNF-alpha blocking agent, golimumab administered subcutaneously (50, 100 or 200 mg) once a month or placebo, there was no significant difference in clinical efficacy between the 4 groups [64]. There was a higher frequency of severe adverse reactions, including serious and life-threatening infections in the golimumab-treated groups compared with placebo. One death and all 8 malignancies occurred in the golimumab-treated groups. The steering committee decided to discontinue the study-agent administration after reviewing the safety data at the Week-24 database lock.

The relatively small patient populations studied in these trials makes it difficult to assess the safety of TNF-alphas blocking agents in asthma. However, these agents have been extensively studied in rheumatologic diseases. In an open-label extension study of 257 patients with ankylosing spondylitis treated for up to 192 weeks with etanercept, injection site reactions, diarrhea and headaches were the most frequent complaints [65]. The rate for serious infections, which is listed in a 'black-box' labeled warning in the package insert, was 0.02 per patient-year. The safety of TNF-alpha blocking agents was evaluated in an open, prospective study of 163 patients with juvenile idiopathic arthritis: 68 received infliximab and 95 received etanercept [66]. Adverse leading to discontinuation occurred in 26 (32.1%) patients treated with Infliximab and 18 (14.2%) patients treated with etanercept. The authors noted that some AEs, "..such as thrombocytopenia, neuro-psychiatric disorders, new onset of Crohn's disease, new onset or flare-up of chronic iridocyclitis, were unusual and had rarely been described before, yet proved to be significant in frequency and/or clinically noteworthy in the large population.." they followed. One review of 18 randomized trials involving 8800 patients, found no increase in the odds of death, serious adverse events, serious infection, lymphoma, non-melanoma skin cancers or the composite endpoint of non-cutaneous cancers plus melanomas with recommended doses of TNF-alpha blocking agents when evaluated using the unadjusted meta-analytic method [67]. For individuals receiving two to three the times the recommended dose of the TNF-alpha blocking agent, there was a twofold increase in serious infection.

As discussed earlier in the paper there are conflicting data on the risk of malignancies with TNF-alpha blocking agents [4,5]. In August of 2009, the FDA notified healthcare professionals that it has completed its analysis of TNF-alpha blocking agents and concluded that there is an increased risk of lymphoma and other cancers associated with the use of these drugs in children and adolescence [68]. The current prescribing information for TNF-alpha blocking agents does contain a warning for malignancies, but does not specifically mention leukemia. This new safety information is now being added to the *Boxed Warning *for these products.

Other potentials safety issues with TNF-alpha blocking agents include (all type γ effects): [69-71]

•Increased susceptibility to tuberculosis (TB) and certain opportunistic infections or reactivation of latent TB--screening prior to initiation of therapy should include identifying risk for latent TB (e.g., HIV infection, drug addiction, living in a region of high TB prevalence, etc.), tuberculin skin test and chest x-ray [70]

•Serious bacterial infections: incidence of 0.07 to 0.09 per patient year compared 0.01 to 0.06 per control population year [70]

•Autoimmune- like syndromes: lupus-like reactions, demyelinating syndromes

## Conclusion

There are several reasons why biologic agents may offer distinct advantages over conventional pharmacotherapy in the treatment of rhinitis and asthma. Both conditions are likely to present with active symptoms requiring some medical management for many years [72]. Biological agents that have an immunomodulatory effect such as allergen immunotherapy may produce sustained clinical improvement after discontinuation of treatment, Whereas, conventional pharmacotherapy is generally only effective during active treatment [3]. The most serious adverse reaction from biologic agents is anaphylaxis and this has been seen most frequently seen in SCIT with unmodified extracts. The delivery of these unmodified allergens via the sublingual route appears to be associated with less risk of anaphylaxis and similar efficacy.

Newer immunotherapy formulations containing Th1 adjuvants (CpG and MPL) appear to provide the benefits of SCIT with a much shorter course of treatment (4 to 6 injections) and a lower risk of adverse events. Biologic agents that target specific components of the immune system may provide clinical efficacy to patients that have failed to respond to optimal pharmacotherapy. Omalizumab is the most extensively studied and prescribed agent for the treatment of allergic asthma that targets a single molecule. Adverse reactions to omalizumab are uncommon but anaphylaxis (none fatal) has been reported at a rate of 0.09 to 0.2% of patients. Some studies have suggested that patients on omalizumab, who are at high risk of geohelminth infections, may be more susceptible to parasitic infection but not more resistant to treatment. Other biologic agents targeting IL-5, and IL-4/IL-13 did not demonstrate any serious or severe treatment-related adverse events in asthma patients, There has been some conflicting information on the safety of TNF-alpha blocking agents in the treatment of asthma.

In general, these biological agents were studied in clinical trials that included relatively small patient populations and there may have not been a sufficient number of patients to identify adverse treatment-related events that occur at a low frequency. Some uncommon adverse effects, such as 1 fatality in 2.5 million SCIT injections or anaphylaxis in 0.09% of omalizumab-treated patients, may not become apparent in clinical trials or postmarketing surveillance data until a very large number of patients have been studied or the medication has been in use for several years. In general, the risk of serious reactions, such as anaphylaxis, with these biological agents is relatively small, and may be reduced by appropriate medical supervision during treatment.
